# Association Between Single Nucleotide Polymorphisms in DNA Polymerase Kappa Gene and Breast Cancer Risk in Chinese Han Population: A STROBE-Compliant Observational Study: Erratum

**DOI:** 10.1097/01.md.0000481302.82879.cc

**Published:** 2016-03-03

In the article “Association Between Single Nucleotide Polymorphisms in DNA Polymerase Kappa Gene and Breast Cancer Risk in Chinese Han Population: A STROBE-Compliant Observational Study”,^1^ which appeared in Volume 95, Issue 2 of *Medicine*, the Ki67 index value was originally misrepresented in several locations throughout the text. The article has since been corrected online.
